# Neuropeptides regulate embryonic salivary gland branching through the FGF/FGFR pathway in aging klotho‐deficient mice

**DOI:** 10.1111/acel.14329

**Published:** 2024-09-06

**Authors:** Nguyen Khanh Toan, Soo‐A Kim, Sang‐Gun Ahn

**Affiliations:** ^1^ Department of Pathology, School of Dentistry Chosun University Gwangju Republic of Korea; ^2^ Department of Biochemistry, School of Oriental Medicine Dongguk University Gyeongju Republic of Korea

**Keywords:** accelerated aging, branching morphogenesis, klotho, neuropeptides, salivary gland

## Abstract

Salivary gland branching morphogenesis is regulated by the functional integration of neuronal signaling, but the underlying mechanisms are not fully understood in aging accelerated klotho‐deficient (Kl^−/−^) mice. Here, we investigated whether the neuropeptides substance P (SP) and neuropeptide Y (NPY) affect the branching morphogenesis of embryonic salivary glands in aging Kl^−/−^ mice. In the salivary glands of embryonic Kl^−/−^ mice, morphological analysis and immunostaining revealed that epithelial bud formation, neuronal cell proliferation/differentiation, and the expression of the salivary gland functional marker ZO‐1 were decreased in embryonic ductal cells. Incubation with SP/NPY at E12‐E13d promoted branching morphogenesis, parasympathetic innervation, and epithelial proliferation in salivary glands of embryonic Kl^−/−^ mice. The ERK inhibitor U0126 specifically inhibited neuronal substance‐induced epithelial bud formation in the embryonic salivary gland. RNA‐seq profiling analysis revealed that the expression of fibroblast growth factors/fibroblast growth factors (FGFs/FGFRs) and their receptors was significantly regulated by SP/NPY treatment in the embryonic salivary gland (E15). The FGFR inhibitor BGJ389 inhibited new branching formation induced by SP and NPY treatment and ERK1/2 expression. These results showed that aging may affect virtually the development of salivary gland by neuronal dysfunction. The neuropeptides SP/NPY induced embryonic salivary gland development through FGF/FGFR/ERK1/2‐mediated signaling.

## Graphical abstract

Schematic representation of Neuropeptide SP/NPY pathways regulating sallvary gland morphogenesis/development. SP/NPY induced ambryonic sallvary gland development through FGF/FGFR/ERK1/2‐mediated signalling.

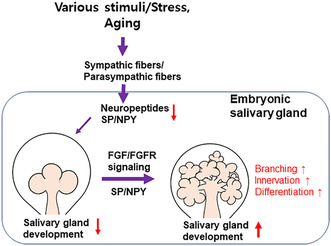

**Abbreviations**
AQP5: aquaponrin 5
cAMP: cyclic adenosine monophosphate
ERK1/2: extracellular signal‐regulated kinase 1/2
FGFRs: fibroblast growth factor receptors
FGFs: fibroblast growth factors
Kl: klotho
Krt: keratin
mTOR: mammalian target of rapamycin
NPY: neuropeptide Y
SP: substance P
ZO‐1: zonula occludens‐1

## 1 INTRODUCTION

The salivary gland is an essential component of human daily activity and plays a crucial role in food digestion and the maintenance of oral health (Chibly et al., 2022). The main roles of salivary glands include producing and secreting saliva, a fluid that participates in multiple roles, including initiating food digestion, maintaining antibacterial activity, protecting oral structures, and assisting in speaking and articulation through lubrication (Chibly et al., 2022; Pedersen et al., 2018). Loss of saliva production, also known as hyposalivation, can lead to painful and devastating oral conditions and deterioration of oral health, including difficulty eating, increased dental decay, disrupted oral homeostasis, increased oral disease, and periodontitis (Rocchi & Emmerson, 2020). Hyposalivation usually occurs in head and neck cancer patients as a side effect of radiotherapy treatment, as a result of autoimmune diseases such as Sjögren's syndrome, or from the side effects of medication or due to the natural aging process (Chibly et al., 2022; Rocchi & Emmerson, 2020; Xu et al., 2019). Compared with those of younger people, aged salivary glands exhibit acinar atrophy, ductal dilation, reduced blood vessel density, and increased deposition of fibro‐adipose tissue (Scott et al., 1987). Additionally, there is an increase in immune response signals and heightened infiltration of inflammatory cytokines, along with a decrease in mitochondrial numbers and impaired oxidative phosphorylation (Li et al., 2023). All these alterations lead to a reduction in saliva production. Despite substantial research on xerostomia and dry mouth syndrome treatments, the development of new therapies has been limited, as evidenced by numerous clinical trials yielding minimal outcomes and no permanent solution available for restoring damaged acinar cells in the salivary gland (Rocchi & Emmerson, 2020).

The innervation of the salivary gland has been suggested to be involved during both the development and physiological function of salivary glands (Knox et al., 2010; Proctor & Carpenter, 2007). The salivary gland receives autonomic innervation from both sympathetic and parasympathetic nerves (Teshima et al., 2019). During the development, parasympathetic innervation maintained the progenitor cell population at the periphery of the epithelial buds and influenced the lumenization through the cAMP signaling pathway (Knox et al., 2010; Nedvetsky et al., 2014). Sympathetic innervation occurs at embryonic day 15.5 in murine, when the catecholaminergic nerves from the superior cervical ganglion (Ferreira & Hoffman, 2013; Teshima et al., 2019). Inhibition of catecholaminergic neurons impaired gland formation, however, the majority of their roles remain undiscovered (Teshima et al., 2019). In the matured glands, parasympathetic signal promotes vasodilation and fluid secretion via neurotransmitter acetylcholine and substance P (SP), whereas norepinephrine targets sympathetic nerves to induce the vasoconstriction and secretion of mucous saliva (Ekström, 1989). Both types of innervation can also act directly on secretory cells, resulting in increased levels of intracellular Ca^2+^, changes in membrane permeability, and the secretion of saliva (Chibly et al., 2022; Proctor & Carpenter, 2007). In addition, neuropeptides/neurotransmitters released from nerve fibers also stimulate adrenergic and/or cholinergic neurotransmitter receptors to stimulate acinar and duct cells. This suggests that neural regulation controls salivary gland function. However, less is known about salivary gland innervation during development and how developing nerves influence salivary gland organogenesis.

Neuropeptides are small proteins secreted by neurons that mostly bind to G protein‐coupled receptors to stimulate long‐term effects in the nervous system and thus impact the majority of innervated organs (Russo, 2017). The beneficial impacts of neuropeptides in the salivary gland vary from basic functions, such as stimulating/inhibiting protein and fluid secretion, to complicated functions, such as modulating the immune response, facilitating salivary gland development, or being involved in feeding behavior (Aras & Ekström, 2006; Bauer et al., 1989; Lee et al., 2017; Nedvetsky et al., 2014; Sato et al., 2020). Conversely, several neuropeptides can inhibit salivation, such as opioids (Bruera et al., 1999). Recent research showed that the SP and neuropeptide Y (NPY) on salivary glands reveal significant interactions that influence glandular function and salivary secretion. Some studies have demonstrated that SP can stimulate the glandular secretory functions by acting on neurokinin in the salivary glands (Virta et al., 1994). Additionally, SP increased the weight of the salivary glands and the rate of salivary‐gland cell proliferation (Del Fiacco et al., 2015; Horie et al., 1996). Neuropeptide Y also plays a critical role in various physiological processes, including the regulation of salivary secretory function (Dawidson et al., 1997; McCloskey & Potter, 2000). Previous studies reported the mechanisms through which NPY influences salivary glands, highlighting its effects on salivary protein secretion and Ca^2+^ channel regulation (Ekström et al., 1996; Endoh et al., 2012). Interestingly, SP and NPY can have both synergistic and antagonistic effects on salivary glands. For instance, while SP primarily promotes inflammatory responses, NPY may counteract these effects through its anti‐inflammatory properties. Conversely, their combined action on blood flow and secretion can complicate the overall impact on glandular function (Bedoui et al., 2003; Ferreira & Hoffman, 2013). Understanding the interactions between neuropeptides (SP and NPY) in salivary glands provides a potential therapeutic method. Targeting these neuropeptides could help manage conditions like aging‐related disorders (dry mouth), inflammatory diseases, and fibrosis of the salivary glands.

These neuropeptides are also capable of inducing protein and fluid secretion in the salivary gland and possess great potential for regenerative medicine (Chibly et al., 2022). In addition, it has been reported that NPY and SP were successfully applied in cardiovascular regeneration, wound healing, neurogenesis, and antiaging (Barbariga et al., 2018; Decressac et al., 2011; Michalkiewicz et al., 2003; Park et al., 2007). Therefore, in this study, we hypothesized that the neuropeptides NPY and SP are involved in embryonic salivary gland development and evaluated the potential impact of these neuropeptides using an ex vivo embryonic salivary gland culture system.

Previous studies suggested that the klotho protein could play a crucial role in regulating the aging process. In animal models, increased levels of klotho protein have shown extended lifespan and a reduced incidence of age‐related diseases, indicating that klotho protein can play a significant role in suppressing pathological processes associated with aging (Kuro‐o et al., 1997; Kurosu et al., 2005). Klotho‐deficient mice exhibit premature aging symptoms, experiencing a range of aging‐related diseases such as short lifespan, thinning of the skin, growth retardation, osteoporosis, infertility, increased oxidative stress, and cognitive impairment (Kuro‐o et al., 1997). Therefore, the klotho‐deficient mice model has made important contributions to understanding the aging process and developing new strategies for preventing or treating age‐related diseases. Using aging accelerating klotho‐deficient mice, our studies will continue to assist in deepening our understanding of the complex mechanisms of aging and finding new ways to regulate aging.

In this study, we demonstrated that embryonic salivary gland morphogenesis is inhibited in an aging mouse model and that the expression of neuronal and ductal proteins is decreased. Treatment with the neuropeptides NPY and SP promoted branching morphogenesis, neurogenesis, and epithelial proliferation in the embryonic salivary gland in animal models of aging. We also found that the development of embryonic salivary glands by the neuropeptides NPY and SP is induced through the FGF/FGFR/ERK1/2 signaling pathways, as demonstrated through loss‐of‐function experiments. Our results provide novel insight into the potential function of the neuropeptides NPY and SP in the development of the embryonic salivary gland.

## 2 RESULTS

### 2.1 Embryonic salivary gland development in Kl^−/−^ mice undergoing accelerated aging

**FIGURE 1:**
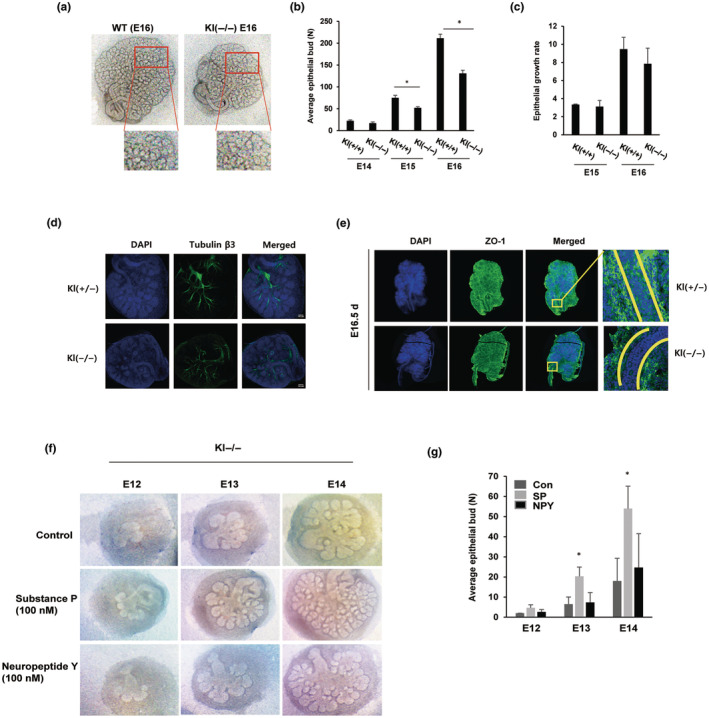
Aging disrupts branching morphogenesis in embryonic salivary glands ex vivo (a) Embryonic salivary glands from wild type (WT) and klotho knockout (Kl^−/−^) mice were isolated at the bud stage (E12) and placed on the surface of polycarbonate track‐etched membrane filters for culture at the air–liquid interfaces from 2 days to 4 days. Brightfield images were taken every 24 h. (b, c) Quantification of epithelial bud formation or the epithelial growth rate of embryonic salivary gland formation at the pseudoglandular stage (E13.5) or canalicular stage (E16) in WT and aging Kl^−/−^ mice. *p < 0.05. (d) Immunofluorescence staining for the parasympathetic nervous system marker tubulin β3 and the ductal marker ZO‐1 at the canalicular stage in WT and aging Kl^−/−^ embryonic salivary glands using confocal microscopy. Representative images of tubulin β3 and ZO‐1 (green) and DAPI (blue) immunostaining are shown. (f, g) Effects of neuropeptides SP and NPY on ex vivo branching morphogenesis. Embryonic salivary glands from Kl^−/−^ mice were harvested at E12 and cultured ex vivo for 48 h. 100 nM of neuropeptides SP or NPY was added to the culture media and then refreshed every 24 h. (f) Brightfield images of embryonic salivary glands during 48 h of neuropeptide treatment. (g) Quantification of the number of embryonic salivary gland end buds at different time points (*n* = 3; *p < 0.05).

Salivary gland branching morphogenesis is a complex process that requires the cooperation of numerous signaling factors. We adapted ex vivo culture of embryonic salivary glands to adequately mimic this process and to analyze the morphogenesis of the salivary gland during organogenesis. As shown in Figure 1a, the number of epithelial buds in the salivary glands was markedly lower in Kl^−/−^ mice than in wild‐type (WT) mice. Quantification of epithelial buds revealed that at the initial timepoint (E14), the WT mice had a slightly greater number of epithelial buds than did the Kl^−/−^ mouse samples. However, the difference in epithelial bud number between WT and Kl^−/−^ embryonic salivary glands became more pronounced in a time‐dependent manner. On E15, WT had 44% more epithelial buds than Kl^−/−^ mice; and on E16, WT mice showed a higher number of epithelial buds (approximately 62%) than the Kl^−/−^ mice (Figure 1b). Interestingly, the epithelial growth rate was not significantly different between WT and Kl^−/−^ mice (Figure 1c). The results of the immunofluorescence staining assay for the neuronal marker β‐tubulin III revealed that the innervation of cholinergic neuronal cells in salivary glands was reduced in Kl^−/−^ embryonic salivary glands compared to WT salivary glands (Figure 1d). At E16, expression of ZO‐1, a marker of ductal differentiation, was also significantly reduced in the lumen of the main duct of the Kl^−/−^ embryonic salivary gland (Figure 1e).

To examine the potential effect of neuropeptides on salivary gland branching morphogenesis, embryonic salivary glands were isolated and cultured in an organ culture medium supplemented with SP or NPY for 48 h. Treatment with SP significantly promoted branching development in the embryonic salivary gland, as demonstrated by the increase in the number of epithelial buds (Figure 1f). Conversely, compared with the control treatment, NPY treatment also induced branching development, but the difference was not significant (Figure 1g).

### 2.2 The neuropeptides NPY and SP induce branching morphogenesis, neurogenesis, and epithelial cell proliferation in the embryonic salivary gland

**FIGURE 2:**
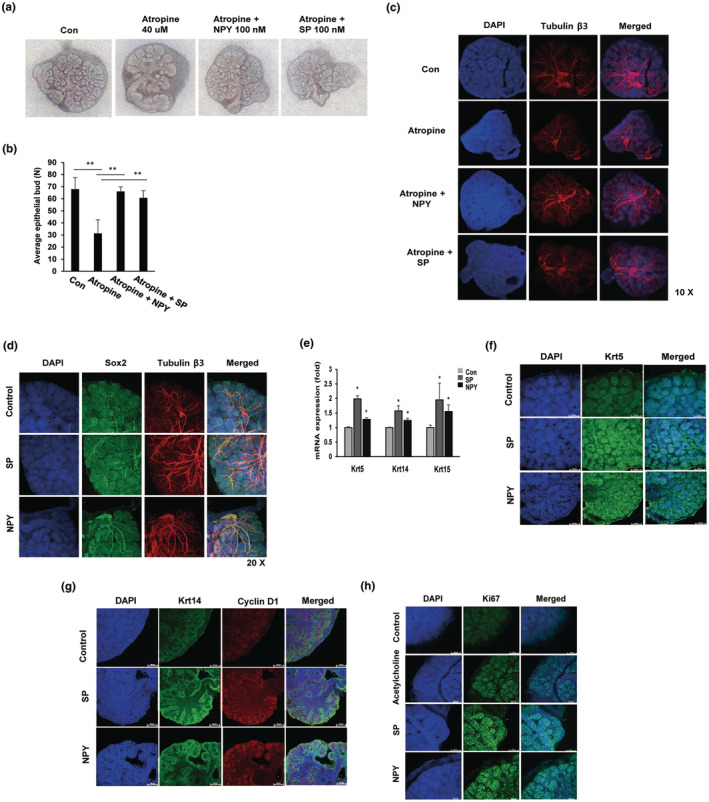
The parasympathetic nervous system participates in branching morphogenesis and neurogenesis in embryonic salivary glands. The neuropeptides SP and NPY induce neurogenesis in the presence of the cholinergic inhibitor atropine. Embryonic salivary glands of the wild type were harvested at E12.5 and then cultured with the neuropeptides SP/NPY and/or the cholinergic inhibitor atropine for 48 h. (a) Brightfield images of embryonic salivary glands at the pseudoglandular stage (E14). (b) Quantification of the number of epithelial buds in the embryonic salivary gland (*n* = 3, **p < 0.01). (c) Immunostaining of the parasympathetic nervous system marker tubulin β3 at the pseudoglandular stage (E14). (d) The effect of neuropeptides SP and NPY on embryonic salivary gland stem cells. Embryonic salivary glands (E13.5) were treated with 100 nM NPY or SP for 48 h and then immunostained for tubulin β3 and Sox2, stem cell markers, using confocal microscopy. 20× magnification. (e, f) The effects of SP and NPY on epithelial cell proliferation (e) After SP or NPY treatment for 48 h, the expression of epithelial cell markers, such as Krt5 (epithelial progenitors), Krt14 (basal epithelial cells), and Krt15 (intermediate epithelial cells), was assessed via quantitative real‐time PCR. *p < 0.05. (f, g) The embryonic salivary glands were immunostained for Krt5, Krt14, and cyclin D1. 20× magnification. (h) Immunofluorescence staining for Ki67 was used to evaluate cell proliferation. 20× magnification. FGFR, Fibroblast growth factors and their receptors; NPY, neuropeptide Y; PCR, polymerase chain reaction; SP, substance P.

To examine the potential effects of SP and NPY on the parasympathetic nervous system in embryonic salivary glands, we used the cholinergic inhibitor atropine. As demonstrated in Figure 2a, cholinergic inhibition by atropine significantly abrogated salivary gland branching morphogenesis; however, supplementation with the neuropeptide SP or NPY rescued the atropine‐induced reduction in epithelial branching, as demonstrated by the quantification of the number of epithelial buds in Figure 2b. On the basis of the confocal microscopy analysis, immunostaining for the neuronal marker tubulin β3 confirmed a reduction in parasympathetic innervation in atropine‐treated embryonic salivary glands. The neuropeptides SP and NPY induce innervation in atropine‐treated embryonic salivary glands (Figure 2c).

To confirm the involvement of the neuropeptides NPY and SP in neurogenesis during salivary gland morphogenesis, we isolated embryonic salivary glands (E13.5) and treated them with neuropeptides for 48 h. Immunofluorescence staining was performed for Sox2, a stem cell marker, and Tubb3. As demonstrated in Figure 2d, Sox2 was strongly expressed in the innervation of the embryonic salivary gland and colocalized with tubulin β3. We also observed clearly extensive neuron formation at a higher magnification compared with the control (Figure S1a).

To determine whether neuropeptides affect epithelial cell proliferation in embryonic salivary glands, immunofluorescence staining was performed. Cytokeratin 5 (Krt5) and cytokeratin 14 (Krt14) were established as markers of progenitor cells in the basal layer of the epithelium. Cytokeratin 15 (Krt15) is a marker for both inner and outer ductal cell clusters. We found that the mRNA expression of Krt5, Krt14, and Krt15 was significantly induced after 48 h of treatment with NPY or SP (Figure 2e). In addition, the qRT‐PCR results for Krt5, Krt14, and Krt15 were verified via immunofluorescence staining. We showed that the neuropeptides NPY and SP induced greater expression of Krt5 and Krt15 in the peripheral area of the epithelial buds than in the untreated embryonic salivary glands (Figure 5f,g). Additionally, the expression of cyclin D1, a marker of cell cycle progression, was also upregulated after neuropeptide treatment and colocalized with Krt14. We also found that Krt15‐positive cells show upregulated expression after 48 h of incubation with NPY/SP in the roots of the main ducts of the embryonic salivary glands (Figure S1b). Finally, staining for the proliferation marker Ki67 also verified that both the neuropeptides NPY and SP induced epithelial cell proliferation at peripheral epithelial buds (Figure 2h). These results suggest that the neuropeptides NPY and SP promote epithelial cell proliferation in embryonic salivary glands.

### 2.3 NPY and SP induce functional activity in embryonic salivary glands and inhibition of the ERK1/2 signaling pathway abrogated NPY‐/SP‐induced branching morphogenesis

**FIGURE 3:**
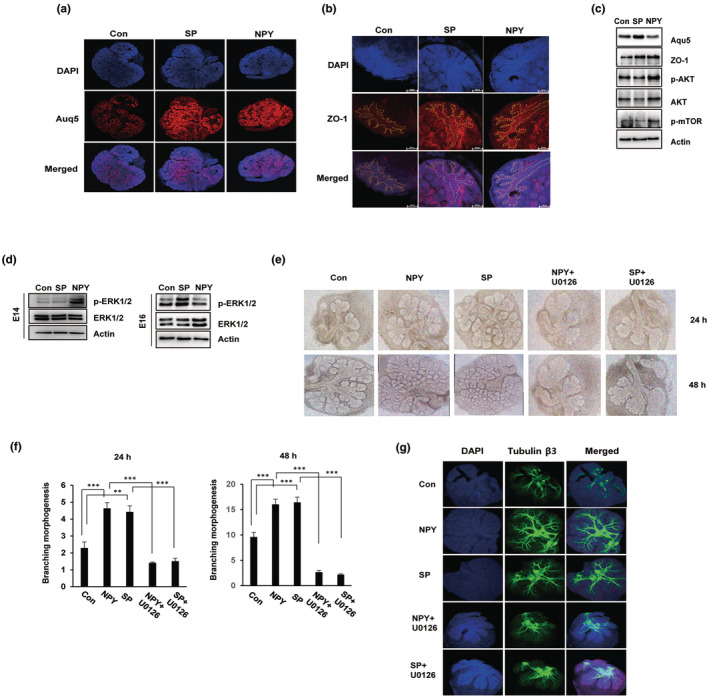
SP and NPY induce functional activity in embryonic salivary glands. Embryonic salivary glands were harvested at the canalicular stage (E15.5) from wild‐type mice and were treated with SP or NPY for 48 h. (a, b) Immunofluorescence staining for the acinar marker AQP5 and the ductal marker ZO‐1 was performed, and images were captured using a confocal microscope. Dash indicates duct. (c) Western blot analysis of E17 embryonic salivary glands treated with the corresponding neuropeptides. The expression of AQP5, ZO‐1, p‐AKT, and p‐mTOR in the SP‐ and NPY‐treated salivary glands was examined by Western blotting; Actin was used as a loading control. (d) The ERK signaling pathway on branching morphogenesis and neurogenesis. Western blotting of total cell lysates isolated from embryonic salivary glands. Isolated embryonic salivary glands were stimulated for the indicated times with SP and NPY. Phosphorylation of p44/42 ERK in response to SP or NPY is induced at the early or late stage, respectively, of embryonic salivary gland development. (e) Brightfield images of branching morphogenesis in neuropeptide‐treated embryonic salivary glands with or without the ERK1/2 inhibitor U0126. (f) Quantification of epithelial buds in neuropeptide‐treated embryonic salivary glands at the indicated times (*n* = 10, **p < 0.01, ***p < 0.01). (g) Immunofluorescence staining of the parasympathetic nervous system marker tubulin β3 in embryonic salivary glands using confocal microscopy. NPY, neuropeptide Y; SP, substance P.

To further investigate the potential effects of NPY and SP on salivary gland function, embryonic salivary glands (E15.5) were harvested and treated with neuropeptides for 48 h. Then, the embryonic salivary glands were stained for the secretory protein AQP5, a marker of acinar maturation, and the tight junction protein ZO‐1, a marker of luminal development during the canalicular stage. As shown in Figure 3a, AQP5 was strongly induced by treatment with SP or NPY. Similarly, the expression of the tight junction protein ZO‐1 was also upregulated in the ductal lumen of salivary glands treated with NPY and/or SP, and the size of the lumen was broader than that of untreated embryonic salivary glands (Figure 3b). Western blotting was also used to determine the potential proteins involved in salivary gland functional activity. Substance P induced the expression of AQP5 and ZO‐1 in embryonic salivary glands but did not induce the activation of Akt/mTOR. However, NPY is involved in ZO‐1 expression and induces the phosphorylation of Akt and mTOR (Figure 3c). These data suggest that the neuropeptides NPY/SP induce salivary gland functional activity through an Akt/mTOR‐dependent or Akt/mTOR‐independent pathway in the canalicular stage of the embryonic salivary gland.

A previous study suggested that NPY and SP can each activate the ERK1/2 signaling pathway; therefore, we investigated the impact of the ERK1/2 signaling pathway on neuropeptide‐induced epithelial branching morphogenesis in embryonic salivary glands. Western blot analysis revealed that ERK1/2 phosphorylation is activated by treatment with NPY or SP at the early stage (pseudoglandular and canalicular stage, E14) and late stage (acinar/lumen maturation, or E16), respectively (Figure 3d). Cotreatment with neuropeptides and the ERK1/2 inhibitor U0126 significantly inhibited NPY‐/SP‐induced branching morphogenesis in embryonic salivary glands (Figure 3e). In particular, compared with NPY‐ or SP‐treated embryonic salivary glands, the ERK1/2 inhibitor U0126 inhibited epithelial growth and peripheral bud formation; in the presence of U0126, the epithelial growth rate was reduced by 66% after 24 h and 85% after 48 h compared with that in the neuropeptide‐treated embryonic salivary glands (Figure 3f). In addition, immunofluorescence staining for the neuronal marker tubulin β3 showed that nervous system development was also inhibited by the presence of an ERK1/2 inhibitor. In the NPY/SP‐treated embryonic salivary glands, the nervous system expanded greatly to the peripheral buds, while in the embryonic salivary glands treated with U0126, the nervous system was mostly localized around the main ducts, and the formation of new nerve branches was inhibited (Figure 3g).

### 2.4 RNA‐sequencing analysis of NPY‐/SP‐induced branching morphogenesis

To identify new neuropeptide‐specific and/or tissue regulators, we performed RNA‐seq‐based expression profiling of the mouse embryonic salivary glands at early stages of embryonic development. We isolated total RNA from embryonic salivary glands dissected from E13.5 embryos (pseudoglandular stage) and then treated them with neuropeptides for 48 h.

**FIGURE 4:**
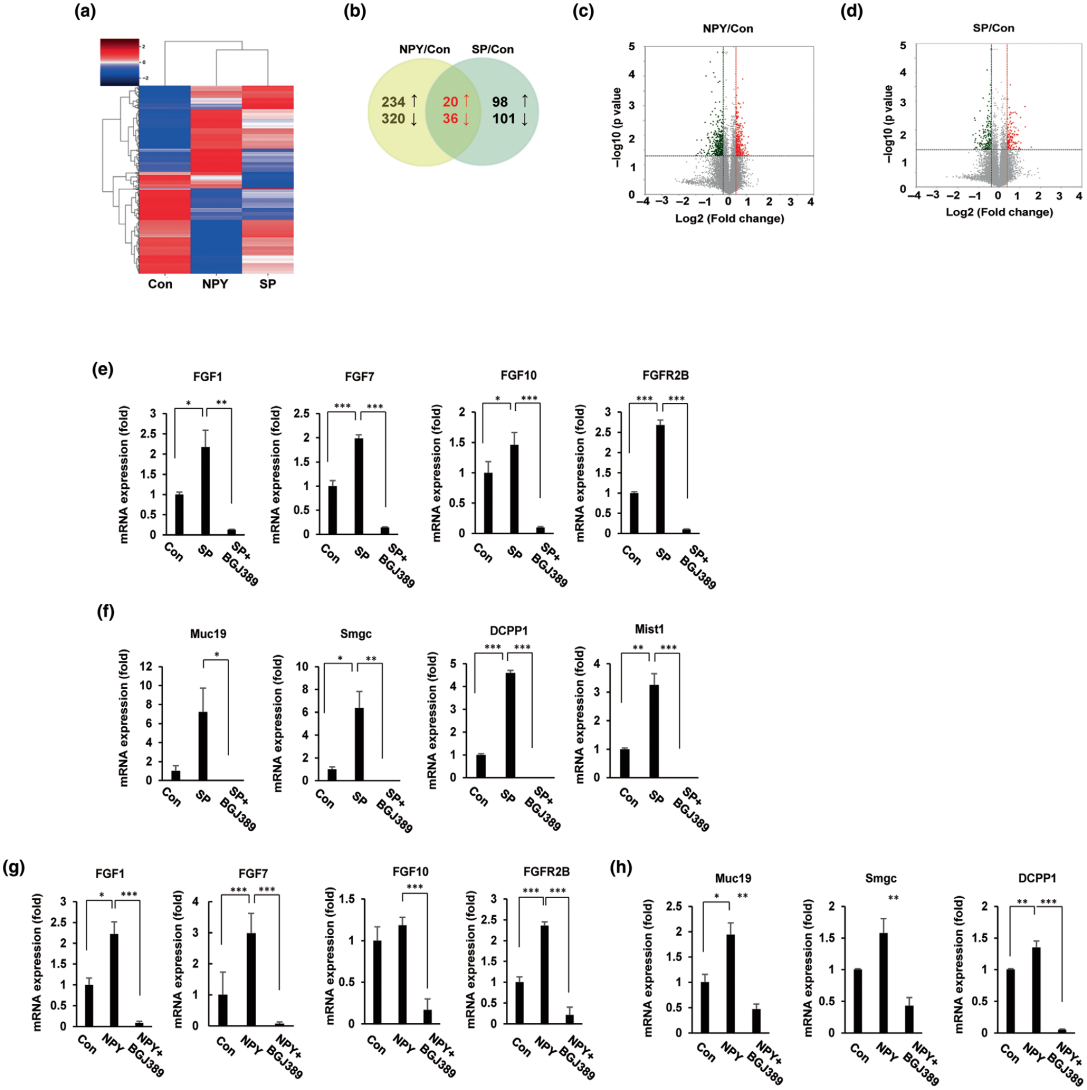
RNA‐seq profiling of branching morphogenesis in neuropeptide‐treated embryonic salivary glands. We isolated total RNA from embryonic salivary glands and performed RNA‐seq. The proportion of variance in each principal component (PC1, PC2, and PC3) represented ~90% of the variance in the data. Utilizing these data sets, we defined and annotated the embryonic salivary gland transcriptional landscape by using various Gene Ontology (GO) annotation analyses. (a) Clustering heatmaps displaying differentially expressed genes (DEGs) between the control group and the neuropeptide NPY‐ or SP‐treated group. (b) Venn diagrams illustrating the number of DEGs in the NPY‐ and SP‐treated groups (≤2‐fold, *p* value = 0.05). (c, d) Volcano plots demonstrating the number of DEGs in the NPY‐ and SP‐treated groups. All the figures were generated using ExDEGA software from Ebiogen. (e) SP/NPY activated the FGF/FGFR signaling pathway and acinar secretory proteins. Embryonic salivary glands (E13.5) were cultured ex vivo for 48 h. Embryonic salivary glands were cultured with the neuropeptides SP/NPY and/or BGJ398 (1 μM/mL). qRT‐PCR analysis of mRNA expression in the presence of SP (e, f) and NPY (g, h) (*n* = 3; mean ± SD; Student's *t*‐test. *p < 0.05, **p < 0.01, ***p < 0.001). FGFR, Fibroblast growth factors and their receptors; NPY, neuropeptide Y; SP, substance P.

To evaluate neuropeptide‐dependent differential gene expression patterns, we analyzed our samples after treatment with 2 neuropeptides during salivary gland embryogenesis (E13.5). For this analysis, we selected genes that showed at least a twofold change in expression between each sample while showing an adjusted *p* value of <0.05. Using the z score, the significantly differentially expressed genes were clustered and visualized using a heatmap (Figure 4a). As shown in Figure 4b–d, Venn diagrams and volcano plots were generated to summarize the numbers of genes significantly up‐ and downregulated by neuropeptide treatment. We identified a total of 554 differentially expressed genes (DEGs) between the control and NPY treatment groups; 234 genes were upregulated, and 320 genes were downregulated. A similar comparison of DEGs between the control and SP treatment groups revealed 199 genes. Among these DEGs, 98 were upregulated and 101 were downregulated. A detailed list of the top 25 downregulated and upregulated genes is provided in Tables [Link], [Link]. Gene Ontology (GO) analysis of the neuropeptide‐induced embryonic salivary gland developmental stage‐specific gene profile associated with specific functions indicated the occurrence of distinct biological processes during embryonic salivary gland development. A closer examination of genes that were uniquely expressed during salivary gland development revealed specific enrichment of biological pathways associated with organs, embryonic development, neuronal development, and ATP synthesis; all of these biological processes are associated with salivary gland development (Figure S2a,b). Interestingly, we also observed specific enrichment in both nervous system development and fibroblast growth factor receptor (FGFR) signaling in embryonic salivary glands during SP‐induced embryonic salivary gland development.

Using qRT‐PCR analysis, we evaluated the expression of NPY and SP‐specific receptors (NPY1R, NPY2R, NPY4R, and TAC1R) in the embryonic salivary glands. Interestingly, the qRT‐PCR results showed that the mRNA levels of NPY and SP‐specific receptors did not change in neuropeptide‐treated embryonic salivary glands (Figure S3), similar to the findings of the raw RNA‐seq data (data not shown). It is suggested that NPY and SP regulate embryonic salivary gland development through a potential noncanonical signaling pathway. Therefore, we focused on FGF/FGFR signaling, which is important for salivary gland differentiation. In agreement with our RNA‐seq data, qRT‐PCR was used to detect the expression of FGFs and FGFRs in the embryonic salivary glands. We determined the expression levels of FGF1, FGF7, FGF10, and FGFR2B *in* NPY/SP‐treated embryonic salivary glands, the results of which suggested the involvement of the FGF/FGFR signaling pathway in embryonic salivary gland development (Figure 4e,f). The expression levels of several markers of acinar differentiation, including the secretory genes Muc19, Smgc, and Dcpp1 and the transcription factor Mist1, were also increased (Figure 4g,h). Next, to examine the potential connection between FGF signaling and neuropeptide‐induced embryonic salivary gland development, we cotreated the cells with neuropeptides and a pan inhibitor of the FGF receptor (BGJ398.) We demonstrated that FGF and FGFR2B expression levels were significantly lower in NPY/SP‐treated embryonic salivary glands treated with BGJ398 than in embryonic salivary glands treated with NPY/SP alone. Similarly, the neuropeptide‐induced expression of Muc19, Smgc, and Dcpp1 was significantly downregulated by the inhibition of FGF receptors (Figure 4e–h).

### 2.5 NPY/SP regulated salivary gland branching morphogenesis through FGFR and its canonical downstream pathway

**FIGURE 5:**
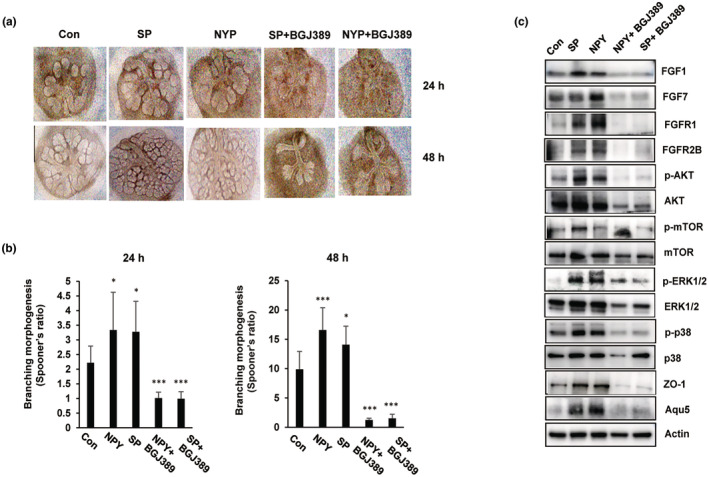
Branching morphogenesis of embryonic salivary glands treated with BGJ398. Embryonic salivary glands were treated with the neuropeptides SP/NPY or BGJ398 for 48 h. (a) Representative images at 24 h and 48 h. Images showing epithelial bud and ductal formation in embryonic salivary glands before/after treatment; these processes were partially inhibited by BGJ398 treatment (*n* = 3/group). (b) Quantification of epithelial buds at the indicated times in neuropeptide‐ and/or BGJ398‐treated embryonic salivary glands. (*n* = 3/group; mean ± SD; Student's *t*‐test. *p < 0.05, ***p < 0.001) (c) Western blot analysis of the expression levels of FGFs/FGFRs and their downstream pathways as well as salivary gland functional proteins. FGF, fibroblast growth factors; FGFR, fibroblast growth factors and their receptors; NPY, neuropeptide Y; SP, substance P.

Therefore, we assessed whether BGJ398 could partially or fully inhibit neuropeptide‐induced salivary gland activity at the start of treatment at E13.5 and then at E15.5. As shown in Figure 5a, representative microscopy images revealed that the formation of epithelial buds in NPY/SP‐treated embryonic salivary glands was significantly inhibited by BGJ398 treatment but not by vehicle treatment. Interestingly, peripheral epithelial buds lost their round shape, and the formation of new buds was strongly inhibited (Figure 5a). Quantification of the epithelial growth rate showed that cotreatment with BGJ398 and neuropeptides reduced the Spooner's ratio by 70% after 24 h and by 90% after 48 h compared with that in the neuropeptide‐treated group (Figure 5b). Next, we investigated the activation/expression of proteins involved in the regulation of FGF/FGFR signaling and salivary gland differentiation (ZO‐1 and AQP5). We found that FGFR1 and FGFR2B, two receptors that are expressed mostly in epithelial structures, are upregulated by treatment with the neuropeptide SP or NPY, as well as the two ligands FGF1 and FGF7. Additionally, downstream proteins of FGFR signaling, including Akt/mTOR, Erk, and p38, are also activated by neuropeptide treatment. Similarly, the protein levels of the acinar secretory marker AQP5 and the lumen structure marker ZO‐1 were also induced by SP or NPY treatment. However, FGFR inhibition by BGJ389 led to a decrease in the expression of the FGF/FGFR signaling pathway and salivary gland function in the embryonic salivary gland (Figure 5c).

### 2.6 FGFR1 and FGFR2 silencing inhibits NPY‐/SP‐induced salivary gland branching morphogenesis

**FIGURE 6:**
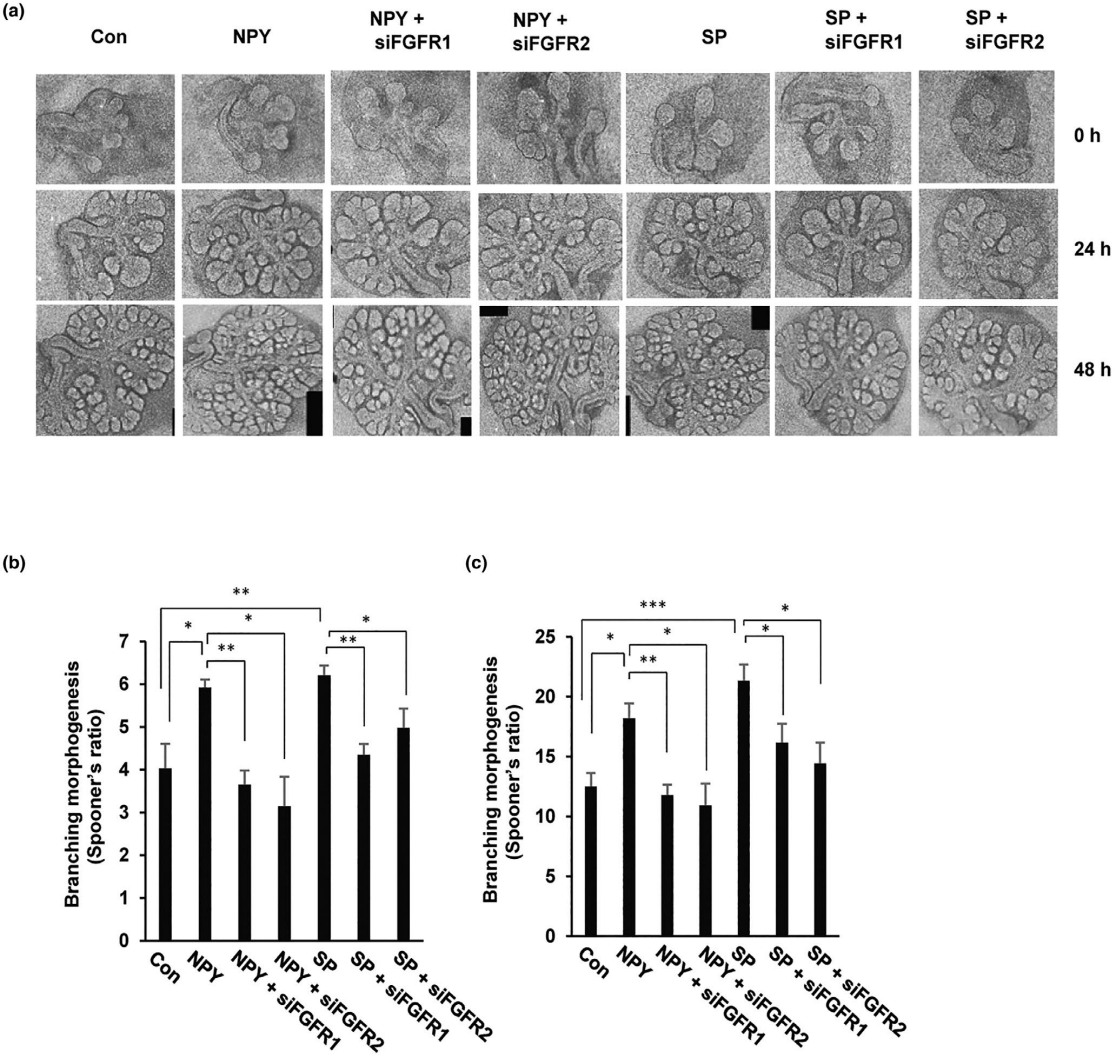
FGFR signaling regulates the branching morphogenesis of embryonic salivary glands. (a) Effects of FGFR1 or FGFR2 siRNA on branching morphogenesis. Light micrographs of E12.5 embryonic salivary glands cultured for 48 h with scrambled control siRNA, siRNA against FGFR1 or FGFR2, or in combination with neuropeptide NPY or SP. (b, c) The graph shows the quantitation of the number of epithelial buds expressed as the number at 24 h and 48 h. *p < 0.05, **p < 0.01, ***p < 0.001. FGFR, fibroblast growth factors and their receptors; NPY, neuropeptide Y; SP, substance P.

Next, we confirmed the effect of the FGF/FGFR pathway on embryonic salivary gland development. For this purpose, we assessed the formation of epithelial buds in embryonic salivary glands treated with siRNAs against FGFR1 and FGFR2. Microscopy analysis revealed that compared with NPY/SP‐mediated epithelial bud formation, treatment with siRNAs against FGFR1 and FGFR2 resulted in a decreased number of epithelial buds in both the pseudoglandular (E13.5) and canalicular (E15.5) stages, suggesting that FGF/FGFR signaling is required for SP‐ or NPY‐induced embryonic salivary gland branching morphogenesis (Figure 6a–c). However, treatment with the control siRNA did not lead to a similar decrease in either stage.

## 3 DISCUSSION

Aging is a complex biological process influenced by genetic, environmental, and lifestyle factors. Previous studies have identified Klotho as an antiaging gene, with its expression linked to the regulation of aging and the lifespan of organisms. Additionally, the roles of klotho in tissue development and maintenance are known well, impacting various organs and systems (Olejnik et al., 2018). In the kidney, Klotho expression is necessary for proper function, and its deficiency is associated with renal aging and pathology. In the brain, Klotho exhibits neuroprotective effects, promoting cognitive function and protecting against neurodegenerative diseases (Li et al., 2004). In our previous studies, klotho‐deficient mouse models have been used to study various facets of salivary gland biology, including physiological function, and stem cell differentiation (Kwon et al., 2018; Tai et al., 2019; Toan et al., 2021). Based on these studies, we have focused on the study of signaling pathways necessary for salivary gland function and embryonic salivary gland development in aging‐accelerated klotho‐deficient (Kl^−/−^) mice.

In this study, we isolated embryonic salivary glands from klotho‐deficient (Kl^−/−^) mice and evaluated embryonic salivary gland morphogenesis and gland function in response to stimulation with the neurotransmitters NPY and SP. Embryonic salivary gland morphogenesis requires complex cellular processes. A network of cellular signaling and regulatory mechanisms is involved in these biological processes and is accompanied by many changes in gene expression throughout embryonic salivary gland development (Suzuki et al., 2021). In mice, there are several distinct developmental stages involved in embryonic salivary gland morphogenesis. Embryonic morphogenesis in the salivary gland starts with localized thickening of the epithelium (early prebud stage [E11]) that progresses to buds and ducts (bud stage [E12.5], pseudoglandular stage [E14], and canalicular stage [E16]) (Suzuki et al., 2021).

During embryonic salivary gland morphogenesis in aging mice, we have shown that morphogenesis is different during the early epithelial bud stage of embryonic salivary development. This stage precedes the active differentiation stage, which is initiated at the canalicular stage (E16). The number of epithelial buds in embryonic salivary glands is reduced in aging Kl^−/−^ salivary glands. In addition, immunostaining analysis revealed a reduction in the expression of the neuronal marker Tubb3 and the ductal marker ZO‐1 in the lumen of the main ducts. A previous study suggested that aging Kl^−/−^ mice exhibit multiple neuronal‐related defects, including synaptic loss, hypomyelination, and neuroinflammation. We first found that neuron formation is inhibited during embryonic salivary gland development in aging Kl^−/−^ mice. We also observed a reduction in the expression of ZO‐1, a salivary gland functional marker, in the salivary glands of Kl^−/−^ mice. Previous studies have highlighted a significant relationship between the tight junction protein ZO‐1 and aging. ZO‐1 is an epithelial marker and a scaffold protein that regulates the movement of ions between endothelial and epithelial cells (Gonzalez‐Mariscal et al., 2008 **)**. During the aging process, expression of ZO‐1 decreased and correlated with an increase of tissue barrier leakage in both humans and mice. These changes are implicated in the pathogenesis of chronic inflammatory diseases, neurodegenerative disorders, and other age‐associated conditions (Goodall et al., 2018). Understanding the molecular mechanisms underlying ZO‐1 degradation and its function could offer new therapeutic targets for mitigating aging‐related diseases. Although we cannot fully understand the development of ductal and epithelial bud structures in Kl^−/−^ mice, aging‐induced pathways are involved in a possible mechanism against embryonic salivary gland development deficiency.

The innervation of the salivary gland has been recently described (Ferreira & Hoffman, 2013). However, the major role of innervation in both the development and physiological function of the embryonic salivary gland in an aging mouse model has not been determined. There are many types of neurotransmitters released by the autonomic nervous system fibers that innervate the salivary glands: acetylcholine (Ach), noradrenaline (NA), vasoactive intestinal peptide (VIP), enkephalin, substance P (SP), neuropeptide Y (NPY), neurokinin A, pituitary adenylate cyclase‐activating peptide, neuronal nitric oxide synthase, and calcitonin gene‐related peptide (CGRP), which provide cholinergic/adrenergic or noncholinergic/adrenergic signaling responses, respectively (Ferreira & Hoffman, 2013). Several studies have explored the relationship between neuropeptides such as substance P (SP) and neuropeptide Y (NPY) with aging (Bannon et al., 2000 **)**. Substance P, known for its role in pain perception and inflammation, has been observed to increase in certain tissues with age, which may contribute to chronic pain and inflammatory conditions commonly seen in older adults (Lauritano et al., 2023). Elevated levels of SP are also associated with neurodegenerative diseases, suggesting its involvement in age‐related cognitive decline (Martinez & Philipp, 2016). Neuropeptide Y, on the other hand, is involved in various physiological processes and neuroprotective effects, and its decline may contribute to the vulnerability of neurons to age‐related damage (Borbély et al., 2013; Pain et al., 2022). Additionally, NPY is linked to several brain functions and behaviors, such as cognition, endocrine regulation, feeding, and the modulation of circadian rhythms, all of which are prevalent in the aging population (Decressac et al., 2012; Thorsell & Ehlers, 2006). Understanding the changes in SP and NPY levels and their impact on aging can provide insights into potential therapeutic strategies to mitigate age‐associated conditions and improve the quality of life in the elderly.

The results of our study demonstrate that the neuropeptides NPY and SP promote branching morphogenesis and functional activity in the salivary glands of aging Kl^−/−^ mice, suggesting that they play roles in promoting salivary gland organogenesis. In particular, our results showed the potential involvement of the neuropeptides NPY and SP in epithelial bud/duct formation. The cholinergic inhibitor atropine inhibited the SP‐/NPY‐induced salivary gland branching morphogenesis. The results showed that NPY and SP are involved in epithelial proliferation and the development of neurons through cholinergic signaling in embryonic salivary glands. A previous study also showed that NPY can facilitate embryonic uretic bud development when co‐treated with GDNF (Choi et al., 2009). Other studies have described several neuronal proteins, including NRTN, NRG1, and VIP, which regulate the lumenization of the salivary gland through the receptor VIP1R and the downstream cAMP/PKA signaling pathway (Knox et al., 2013; May et al., 2022; Nedvetsky et al., 2014). Substance P treatment boosted the proliferation of neural stem cells, induced neural differentiation, and accelerated recovery after ischemic injury (Park et al., 2007; Yang et al., 2017). Similarly, NPY promotes neurogenesis in the dentate gyrus of the hippocampus through its receptor NPY1R and the ERK1/2 signaling pathway both in vitro and in vivo (Decressac et al., 2011). Furthermore, aging reportedly reduces the level of organization of the innervation architecture in the oral cavity and salivary glands (Lahtivirta et al., 1995; Moayedi et al., 2018). A recent study showed that stimulation of parasympathetic nervous system promotes the endogenous regeneration of irradiated acinar cells and the recovery of irradiated salivary glands via functional secretory machines (Li et al., 2022). Using immunofluorescence staining, we also demonstrated that the neuropeptides NPY/SP promote neural branching in the embryonic salivary gland. Together with innervation markers, stem cell markers, including Sox2 are colocalized within the innervation space and are more highly expressed in the outermost area of peripheral buds in the embryonic salivary gland. Our results showed that NPY and SP induce the formation and branching of cholinergic nerves by activating neuronal stem cells in the embryonic salivary gland. Additionally, Sox2 deficiency significantly reduces the number of epithelial buds and prevents the development of acinar cells in the salivary gland, while overexpression of Sox2 can rejuvenate salivary gland tissues that have undergone irradiation injuries (Emmerson et al., 2018). Based on these results, the neuropeptides SP and NPY could potentially help individuals recover from aging‐induced salivary gland dysfunction by promoting neurogenesis. Therefore, we investigated the cellular and molecular mechanisms through which NPY and SP regulate embryonic salivary gland development.

In mammals, there are five receptors for NPY (NPY1R, 2R, 3R, 4R, and 5R), while there is only one receptor for SP (NK1R) (Brothers & Wahlestedt, 2010; Regoli et al., 1994). These G protein‐coupled receptors can trigger multiple signaling pathways, including the MEK/ERK pathway and the PI3K/AKT/mTOR pathway. Specifically, activation of the ERK1/2 signaling pathway was proposed as the mechanism of either NPY‐ or SP‐induced cell recovery. For example, cornea healing was accelerated by activation of ERK signaling after substance P injection (Hong et al., 2009). Treatment of SP activated ERK1/2 signaling to restore functions of bone marrow stem cells originated from osteoporosis rats (Piao et al., 2020). Additionally, NPY exhibited neuroprotective effects under both in vitro and in vivo conditions, also by activating the ERK1/2 mechanism (Decressac et al., 2012). Furthermore, NPY is important and involved in caloric restriction‐induced antiaging through the activation of ERK1/2 signaling (Aveleira et al., 2015; Chiba et al., 2014). In submandibular glands, the MEK/ERK1/2 pathway is also a central signaling pathway that regulates numerous cellular processes, including proliferation, differentiation, and survival, and it participates in the branching morphogenesis of epithelial organs (Kashimata et al., 2000). Our results showed that both NPY and SP can induce ERK1/2 phosphorylation/activation at the pseudoglandular or canalicular stages of embryonic salivary gland development. Consistent with the findings of other studies, pharmacological inhibition of MEK, an upstream effector of ERK1/2, inhibited the epithelial branching of the embryonic salivary gland, even in the presence of NPY/SP (Kashimata et al., 2000). Additionally, we also observed a reduction in innervation in the embryonic salivary gland following treatment with an ERK1/2 inhibitor. These results are consistent with those of several previous studies showing that NPY and/or SP can stimulate neuron proliferation/differentiation through ERK1/2 activation (Decressac et al., 2011; Park et al., 2007; Yang et al., 2017). However, interestingly, we found that the mRNA levels of NPY and SP‐specific receptors did not change in neuropeptide NPY/SP‐treated embryonic salivary glands (data not shown). It is suggested that NPY and SP regulate embryonic salivary gland development through a potentially noncanonical signaling pathway.

We performed a high‐throughput RNA‐seq analysis to identify the potential mechanism through which neuropeptide treatment regulates embryonic salivary gland development in an aging mouse model. RNA‐seq revealed that neuropeptide treatment altered the expression of genes involved in biological processes related to salivary gland organogenesis, such as nervous system development, the cell cycle, the regulation of synapse assembly, and the fibroblast growth factor receptor signaling pathway.

In this study, we found that FGF/FGFR mRNA expression is significantly induced by the neuropeptides NPY/SP in the embryonic salivary gland. Pharmacologically blocking FGFR activation/expression with the pan‐inhibitor BGJ398 inhibited branching morphogenesis and acinar differentiation in NPY/SP‐treated embryonic salivary glands. As mentioned above, RNA‐seq analysis revealed that the neuropeptides NPY/SP induced expression of FGFs/FGFRs and downstream signaling proteins, including ERK1/2, AKT/mTOR, and p38. In addition, Western blot analysis revealed the activation of salivary gland function and the upregulation of AQP5 and ZO‐1 expression following NPY/SP treatment. However, NPY‐/SP‐induced FGF/FGFR downstream signaling was strongly inhibited during cotreatment with BGJ398. Similarly, we demonstrated that siRNA silencing of FGFR1 or FGFR2 decreased NPY‐/SP‐induced branching morphogenesis, suggesting that the neuropeptides NPY and SP are involved in embryonic salivary gland development via the FGF/FGFR signaling pathway. Fibroblast growth factors (FGFs) and their receptors (FGFRs) signaling is necessary for the salivary gland functional effects induced by the neuropeptides NPY/SP.

Fibroblast growth factors (FGFs) and their receptors (FGFRs) signaling and aging have been extensively studied in recent years, revealing complex interactions that influence various aspects of the aging process (Silva‐García, 2023). Fibroblast growth factors (FGFs) and their receptors (FGFRs) play a crucial role in regulating *cellular senescence*, cell growth, differentiation, and survival. Reduced FGF signaling has been linked to impaired wound healing and decreased stem cell function in aged tissues (Ornitz & Itoh, 2022). FGFs, particularly FGF21, are involved in metabolic regulation and have been associated with longevity. FGF21 levels increase with age and have been proposed as a protective factor against age‐related metabolic diseases (Hill et al., 2022; Zhang et al., 2012). In addition, FGF/FGFR signaling is crucial for brain development and function. FGF signaling supports neuronal survival and synaptic plasticity, and its decline with age may contribute to cognitive decline (Guo et al., 2023). However, the precise mechanisms and the balance between the beneficial and detrimental effects of FGF signaling in aging require further investigation.

Previous studies have reported that FGF/FGFR signaling is a key pathway in salivary gland development. Salivary gland hypoplasia with abnormal development was observed in mice genetically lacking FGF10 or the FGFR2IIIb receptor (De Moerlooze et al., 2000; Jaskoll et al., 2005). It has been reported that FGFR pharmacological inhibition or FGF2 knockdown inhibits epithelial pro‐acinar cell differentiation. Additionally, neuropeptide treatment and FGF/FGFR signaling demonstrated synergistic effects on inducing neural cell proliferation in vitro (Rodrigo et al., 2010). NPY administration increases FGFR1 transcription and translation, and SP administration induces the expression of FGF7 in fibroblasts of the cornea (Foldenauer et al., 2012; Rodrigo et al., 2010). Conversely, FGF2 induces the expression of SP and its receptor TAC1R in articular chondrocytes during the inflammatory response (Im et al., 2008). These findings are similar to our results. These results suggest that the neuropeptides NPY/SP promote development in the embryonic salivary gland, partially through interaction with the FGF/FGFR signaling pathway. However, FGFs or FGFRs that directly promote aging‐induced salivary gland dysfunction and neurogenesis have not been identified.

In conclusion, our results provide evidence that in an aging mouse model, branching morphogenesis, neurogenesis, and differentiation are decreased in the embryonic salivary gland. We examined the potential effects and mechanisms of two neuropeptides, NPY and SP, on embryonic salivary gland development in aging Kl^−/−^ mice. These results showed that administration of NPY or SP significantly promoted branching morphogenesis, neurogenesis, epithelial proliferation, and epithelial differentiation through the FGF/FGFR/ERK1/2 signaling pathway during embryonic salivary gland development. Understanding the molecular signaling involved in embryonic salivary gland development during aging can greatly increase our understanding of salivary gland biology and aid in diagnosing the disease state during aging and help to identify potential therapeutic targets for regeneration and tissue engineering approaches in the future.

## 4 MATERIALS AND METHODS

### 4.1 Compounds

The pan‐FGFR inhibitor BGJ398 was purchased from Selleckchem (Houston, TX, USA). The MEK/ERK1/2 inhibitor U0126 was purchased from Promega (Madison, WI, USA). The neuropeptides NPY and SP were purchased from Tocris Bioscience (Bristol, UK). Acetylcholine chloride (Ach‐A6625) was purchased from Sigma (St. Louis, MI, USA). Atropine sulfate (A10236) was purchased from Thermo (Waltham, MA, USA). These compounds were diluted directly into the organ culture medium below the membrane filter, while embryonic salivary glands were cultured on top.

### 4.2 Embryonic salivary gland isolation and culture

Mouse embryonic salivary glands were isolated at embryonic day 13.5 (E13.5). At E13.5, the uteri were removed from the timed pregnant mice and placed in an ice‐cold DMEM/F12 medium. The embryos were removed from their respective uteri using Dumont #5 forceps (Fine Science Tools, 11,251–20). Embryos were placed in a 35‐mm cell culture dish filled with 3 mL of PBS supplemented with P/S prior to salivary gland isolation. Under an Olympus SZ51 dissecting microscope (Olympus Corporation, Tokyo, Japan), the embryos were decapitated using Dumont #5 forceps. The embryonic tails were removed and placed in a 1.5 mL Eppendorf tube for genotype verification. The mandible and the tongue, which surround the salivary gland, were removed from the decapitated head by slicing the forceps across the mouth. The isolated mandible was subsequently placed on the cover of the 35‐mm cell culture dish with the tongue facing down. Subsequently, the prongs of the forceps were slid into the space between the mandible and the tongue, and the midline of the mandible was sliced to expose the tongue and two salivary glands attached to the base of the tongue. After the surrounding tissues were removed, the glands were detached using forceps and collected in a 96‐well plate on ice with 200 μL of DMEM/F12 until all the embryos were dissected. Experiments were performed under approved protocols of the Animal Research Institute Committee of Chosun University for the Care and Use of Laboratory Animals (CIACUC2024‐A0020). Isolated salivary glands were cultured on top of a 0.2 μm nucleopore track‐etched membrane (Whatman, Maidstone, UK, and GVS, Italy) that floated on 1 mL of Organ Culturing Medium in the glass bottom area of the IVF culture dishes (Cat no. 20260, SPL Life Sciences, Gyeonggi, South Korea) at 37°C with 5% CO_2_. The organ culture medium used was DMEM/F‐12 supplemented with 150 mg/mL ascorbic acid (Millipore‐Sigma, St. Louis, MI, USA), 50 mg/mL holo‐transferrin (Millipore‐Sigma, St. Louis, MI, USA), and 1X Pen/Strep (100 units/mL penicillin, 100 mg/mL streptomycin; Thermo Fisher, Waltham, MA, USA).

For compound treatment, NPY, SP, and Ach were diluted in the media to a concentration of 100 nM. The dose of atropine (a cholinergic inhibitor) was 40 μM, while both BGJ398 and U0126 were used at a concentration of 10 μM. The media for embryonic organ culture were replaced every 24 h.

Bright‐field images of isolated embryonic salivary glands were collected every 24 h by an Olympus IX71 (Olympus Corporation, Tokyo, Japan) inverted microscope. The number of epithelial buds was counted using the image processing software FIJI. The epithelial growth rate was calculated as a Spooner ratio using the following equation: the foldchange increase in the epithelial growth rate = the number of epithelial buds at 24 or 48 h/the number of epithelial buds at 0 h.

### 4.3 Genotyping of embryonic salivary gland tissue

We verified the genotypes of the isolated salivary gland samples by performing PCR using genomic DNA isolated from the embryonic tail. Two hundred microliters of tail lysis buffer (0.1 mL of 50 mM Tris (pH 8.0), 100 mM EDTA, 0.5% SDS, and 0.5 mg/mL proteinase K) was added to 1.5 mL Eppendorf tubes containing embryonic tail samples. The tubes were heated at 55°C for 1 h to lyse the whole sample. Two hundred microliters of protein precipitation solution (Promega, Madison, WI, USA) was added to each tube, which was then centrifuged at 12000 rpm at room temperature (RT) for 5 min to pellet the protein. A total of 150 μL of supernatant was collected, and genomic DNA was precipitated by adding the same volume of ice‐cold isopropanol. The mixture was then incubated on ice for 10 min, followed by centrifugation at 12000 rpm at RT for 5 min. The genomic DNA was washed with 400 μL of ice‐cold ethanol (70%) prior to being diluted in 50 μL of DW. The concentration and quality of the isolated genomic DNA were determined using a NanoDrop spectrophotometer DS‐11 (DeNovix, Wilmington, DE, USA).

Genotyping was performed using the polymerase chain reaction (PCR) with two pairs of primers containing a common forward primer and a specific reverse primer for the WT allele or the mutant allele. The expected amplification products were 815 bp and 419 bp for the WT and mutant strains, respectively. Detailed information about the genotyping primers is listed in Table 1. The PCR procedure was as follows: denaturation at 94°C for 5 min; 30 cycles of 94°C for 30 s, annealing at 66°C for 30 s, and extension at 72°C for 45 s; and a final extension at 72°C for 10 min. The PCR products were loaded into 1% agarose gels and visualized under a UV lamp (SL‐20, SeouLin Bioscience, Gyeonggi, Korea).

**TABLE 1:** DNA sequence for primers.

Target	Sequences	Purpose
Rps29	F: 5′‐TCACCAGCAGCTCTACTGGAGT‐3′ R: 5′‐TGAAGCCTATGTCCTTCGCGTA‐3′	qRT‐PCR
FGF1	F: 5′‐GAAAGTGCGGGCGAAGTGTA‐3′ R: 5′‐CATTTGGTGTCTGCGAGCC‐3′	qRT‐PCR
FGF7	F: 5′‐GGCAAAGTGAAAGGGACCCA‐3′ R: 5′‐CAATCCTCATTGCATTCTTTCTTTG‐3′	qRT‐PCR
FGF10	F: 5′‐GTCAGCGGGACCAAGAATGA‐3′ R: 5′‐CGTTGTTAAACTCTTTTGAGCCA‐3′	qRT‐PCR
FGFR2b	F: 5′‐AAGGTTTACAGCGATGCCCA‐3′ R: 5′‐AGAGCCAGCACTTCTGCATT‐3′	qRT‐PCR
Muc19	F: 5′‐CTGGGTCTGGAAGTAGAAGTA‐3′ R: 5′‐TCTAAGCCACAGAAGGAGAT‐3′	qRT‐PCR
Smgc	F: 5′‐TGGCTCTGCAACACAACAGT‐3′ R: 5′‐GGCGAAAAGCTCCCAGGTAA‐3′	qRT‐PCR
Dcpp1	F: 5′‐CGAAACCTCTCAGCCAGACTTT‐3′ R: 5′‐AGTGCAGGAATGTTTTCCAACT‐3′	qRT‐PCR
Mist1	F: 5′‐GCTGACCGCCACCATACTTAC‐3′ R: 5′‐TGTGTAGAGTAGCGTTGCAGG‐3′	qRT‐PCR
Krt5	F: 5′‐CAACGTCAAGAAGCAGTGTGC‐3′ R: 5′‐CAGCTCTGTCAGCTTGTTTCTG‐3′	qRT‐PCR
Krt14	F: 5′‐GCAGCAGAACCAGGAGTACAA‐3′ R: 5′‐CGGTTGGTGGAGGTCACATCT‐3′	qRT‐PCR
Krt15	F: 5′‐ATTCTGGCTGCCACCATTGA‐3′ R: 5′‐GGGTCAGCTCATTCTCATACTTGA‐3′	qRT‐PCR
NPY1R	F: 5′‐CCATCTGACTCTCACAGGCTGT‐3′ R: 5′‐TCTTGTCCATCATGTTGTTTCTCC‐3′	qRT‐PCR
NPY2R	F: 5′‐CGCAAGAGTCAATACAGCCAA‐3′ R: 5′‐CAGAGCAATGACTCTAGGAGTAG‐3′	qRT‐PCR
NPY4R	F: 5′‐CATCTGCCAACCACTCACAGTC‐3′ R: 5′‐CACAGGACCAGTGAGAGGATG‐3′	qRT‐PCR
TAC1R	F: 5′‐AGCTGTGGCCTTTGACAGATAC‐3′ R: 5′‐GCCACTCAATCATGCACACTAC‐3′	qRT‐PCR
Klotho WT	F: 5′‐TTGTGGAGATTGGAAGTGGACGAAAGAG‐3′ R: 5′‐CTGGACCCCCTGAAGCTGGAGTTAC‐3′	Genotyping
Klotho MT	F: 5′‐TTGTGGAGATTGGAAGTGGACGAAAGAG‐3′ R: 5′‐CGCCCCGACCGGAGCTGAGAGTA‐3′	Genotyping

### 4.4 qRT‐PCR

Total RNA was isolated from the pooled embryonic salivary glands (up to 10 samples per condition) using the TaKaRa MiniBEST Universal RNA Extraction Kit (Takara Bio, Inc., Shiga, Japan) following the manufacturer's protocol. In brief, pooled embryonic salivary glands were lysed in 600 μL of extraction solution +5% DTT by pipetting, and then, genomic DNA was removed by running the lysis solution through a gDNA Eraser Spin Column (provided by the manufacturer). Total RNA was precipitated by adding 70% EtOH to the flow‐through mixture and then transferred to an RNA spin column (provided by the manufacturer). Total RNA was bound to the silica membrane in the RNA Spin Column and washed extensively to remove excess reagents. DNAse I was applied to remove the remaining genomic DNA, after which the total RNA was eluted from the RNA spin column. The quality and concentration of total RNA were measured by a NanoDrop Spectrophotometer DS‐11 (DeNovix, Wilmington, DE, USA) to ensure that the A260/A280 ratio fell into a range between 1.8 and 2.1.

Total RNA was used as a template for quantitative real‐time PCR (qRT‐PCR) using a GoTaq 1‐Step RT‐qPCR System Kit (Promega, Madison, WI, USA) according to the manufacturer's protocol. The primers used for qRT‐PCR are listed in Table 1. Rps29 was used as a reference gene. Polymerase chain reaction was carried out as follows: 1 cycle of 95°C for 10 min; 40 cycles of 95°C for 20 s, 60°C for 20 s, and 72°C for 20 s; and a melting curve beginning at 60°C and increasing by 1°C every 6 s, with SYBR green fluorescence measured at every interval. The PCR was performed on a qTOWER3 real‐time PCR thermal cycler (Analytik Jena AG, Thuringia, Germany), and cycle threshold results were analyzed using qPCRsoft version 4.0 by Analytik Jena AG (Thuringia, Germany). Changes in gene expression were tested for statistical significance (*p* < 0.05) relative to the control by Student's *t*‐test.

### 4.5 Whole‐mount immunofluorescence staining

Embryonic salivary gland samples were fixed by replacing the organ culture media below the membrane filter with an approximate volume of 10% neutral buffered formalin and then incubated at 4°C overnight with gentle rocking. Samples were collected in a 12‐well plate, washed with PBS (3 × 15 min), and permeabilized by incubating with PBSTx (PBS + 0.2% Triton X‐100) for 1 h at RT with gentle rocking. Following permeabilization, the samples were blocked in 5% normal goat serum diluted in PBSTx for 1 h at RT with gentle rocking (300 μL solution per well). Then, the samples were incubated with primary antibodies diluted in PBSTx +5% normal goat serum for 48 h at 4°C with gentle rocking (300 μL solution per well). After the primary antibody incubation was complete, the samples were washed 3 × 15 min in PBSTx at RT with gentle rocking. After washing, the samples were incubated with labeled secondary antibodies and DAPI diluted in PBSTx +5% normal goat serum for 48 h at 4°C with gentle rocking (300 μL solution per well). After 48 h of incubation, the samples were washed 3 × 15 min in PBS to remove all excess staining reagents prior to mounting.

Before mounting, the samples were submerged in 500 μL of PBS in a single well of a 12‐well plate. The samples were transferred onto a glass microscope slide (Marienfeld Superior, Germany) by pipetting. Excess PBS was removed by pipetting and using paper towels. Forceps were used to separate the samples to circumvent contact. One hundred microliters of Malinol mounting solution (Muto Pure Chemical, Tokyo, Japan) was pipetted on top of the samples before a 22 × 22 mm thick, #1.5 coverslip (Marienfeld Superior, Germany) was placed on top. After all the samples were mounted, the microscope slides were placed in a dark container at RT for 24 h to allow the mounting media to solidify. The samples were subsequently analyzed by a Nikon Eclipse Ti A1 (Nikon, Tokyo, Japan) confocal microscope. Fluorescence images were acquired by z‐stack imaging with a 3.0 μm step size and a 10X objective, unless otherwise stated. After the z‐stack images were acquired, the final images were generated through maximum intensity Z‐projection using NIS‐Elements software from Nikon (Nikon, Tokyo, Japan).

### 4.6 RNA‐seq and data analysis

Total RNA was isolated from the pooled embryonic salivary glands (up to 10 samples per condition) using the TaKaRa MiniBEST Universal RNA Extraction Kit (Takara Bio, Inc., Shiga, Japan) following the manufacturer's protocol. RNA quality was assessed by an Agilent 2100 bioanalyzer using an RNA 6000 Nano Chip (Agilent Technologies, Amstelveen, The Netherlands), and RNA quantification was performed using an ND‐2000 spectrophotometer (Thermo Fisher, Waltham, MA, USA). Next, a QuantSeq 3' mRNA‐seq Library Prep Kit (Lexogen, Vienna, Austria) was used to construct the library for sequencing following the manufacturer's protocol. In brief, 500 ng RNA was reverse transcribed with Oligo‐dT primers. The RNA template was removed after cDNA synthesis was complete. A tagged double‐stranded cDNA library was synthesized from the single‐stranded cDNA with random primers containing an Illumina‐compatible linker sequence. Magnetic beads were used to purify the double‐stranded cDNA library, and then the cDNA library with adapters and barcodes was generated by another round of PCR and magnetic bead purification. High‐throughput sequencing was performed as single‐end 75‐bp sequencing using an Illumina NextSeq 500 (Illumina, Inc., San Diego, CA, USA).

Data analysis of the QuantSeq 3' mRNA‐seq reads was performed by aligning the reads using Bowtie2. Differentially expressed genes were determined based on counts from unique and multiple alignments using coverage in Bedtools. The read count (RC) data were processed using the TMM + CPM normalization method in the EdgeR package within R using Bioconductor. ExDEGA software from Ebiogen was used to analyze the RNA‐seq data and generate the final figures.

### 4.7 Western blotting

For Western blotting, total protein was isolated from the pooled embryonic salivary glands (up to 10 samples per condition). Embryonic salivary gland samples were collected in a 1.5 mL Eppendorf tube, and 200 μL of RIPA buffer containing 1 g/mL phosphatase inhibitor and protease inhibitor was added to the tube. The Eppendorf tubes were then placed in an ultrasonic bath for 5 min to lyse all the embryonic salivary gland tissues. The sample lysates were subjected to SDS–PAGE and transferred to PVDF membranes (Millipore, Burlington, MA, USA), which were blocked with 5% skim milk for 2 h and incubated with primary antibodies diluted in TBST (TBS + 1% Tween 20) overnight at 4°C with gentle rocking. The next day, the PVDF membranes were washed with TBST 3 times for 5 min before they were incubated with the appropriate secondary antibodies for 1 h at RT with gentle rocking. The membranes were subsequently washed with TBST for 3 × 5 min, after which the protein concentration was detected via the chemiluminescence method. Briefly, the membrane was covered with a 1:1 mixture of Immobilon Western Chemiluminescent HRP Substrate (Millipore, Burlington, MA, USA), and protein signals were visualized using an Amersham ImageQuant 800 system (Amersham, UK).

### 4.8 siRNA transfection

Predesigned siRNAs targeting murine FGFR1 (ID: 14182‐1) and FGFR2 (ID: 14183‐1) were purchased from Bioneer (Daejeon, Korea) and transfected into cultured embryonic salivary glands using Lipofectamine RNAiMAX reagent (Invitrogen, Waltham, MA, USA). One microliter of siRNA and 6 μL of Lipofectamine RNAiMAX were diluted in 100 μL of OptiMEM (Thermo Fisher, Waltham, MA, USA) separately; the mixture was mixed together and incubated at room temperature for 5 min. The final complexes were added to the organ culture medium below the floating membrane and were changed every 24 h. Embryonic organs were cultured with the siRNA complex for 48 h prior to downstream analysis.

### 4.9 Statistical analysis

All the experimental results are presented as the means ± SDs of at least three independent experiments. Two‐sided Student's *t*‐tests were used to compare the means of the two groups. Statistical analysis was performed using two‐way ANOVA or one‐way ANOVA followed by Tukey's post hoc test for multigroup comparisons. *p*‐value of <0.05 was considered statistically significant.

## AUTHOR CONTRIBUTIONS

Nguyen Khanh Toan and Soo‐A Kim: Conception, Methodology, Data validation, Writing – original draft, Writing – review and editing. Sang‐Gun Ahn: Conception, Resource, Funding acquisition, Writing – review and editing, and Supervision. All authors contributed to the revision and review of this article.

## FUNDING INFORMATION

This work was supported by the National Research Foundation of Korea (NRF) grant funded by the Korean government (MSIT) (nos. 2021R1A2C200472311, RS‐2023‐00222390).

## CONFLICT OF INTEREST STATEMENT

The authors have no conflicts of interest to declare. All co‐authors have seen and agree with the contents of the manuscript and there is no financial interest to report.

## Supporting information


Table S1.

Table S2.

Table S3.

Table S4.

**Figure S1.** Immunostaining for Sox2/Tubb3 and Krt15. (a) Immunostaining of the parasympathetic nervous marker tubulin β3 and stem cell markers Sox2, using confocal microscopy. 60× magnification of Figure 2d. (b) Immunostaining with Krt15 after neuropeptide treatment.

**Figure S2.** RNA‐seq profiling. (a,b) Gene Ontology enrichment analysis of biological process terms between the NPY‐ and SP‐treated groups versus control samples generated from the DAVID database. All the figures were generated using ExDEGA software from Ebiogen.

**Figure S3.** qRT‐PCR analysis of the specific receptors against NPY and SP. NPY, neuropeptide Y; PCR, polymerase chain reaction; qRT‐PCR, quantitative real‐time PCR; SP, substance P.

## Data Availability

The data that support the findings of this study are available from the corresponding author upon reasonable request.
