# Supplementary material for: Neuropeptides regulate embryonic salivary gland branching through the FGF/FGFR pathway in aging klotho‐deficient mice
Source: Aging Cell. 2024 Sep 6;23(12):e14329. doi: 10.1111/acel.14329 (PMC11634708; doi:10.1111/acel.14329)
Supplement: Supplementary file 1 — Table S1. [file ACEL-23-e14329-s007.docx]

| **Gene symbol** | **Fold change (FC)** | **Description** |
| --- | --- | --- |
| **Brsk2** | **2.181** | **BR serine /threonine kinase 2** |
| **N4bp2l1** | **2.156** | **NEDD4 binding protein 2-like 1** |
| **Rad52** | **2.107** | **RAD52 homolog, DNA repair protein** |
| **Fbxl8** | **2.104** | **F-box and leucine-rich repeat protein 8** |
| **Wnt2b** | **2.048** | **wingless-type MMTV integration site family, member 2B** |
| **Ap5b1** | **1.984** | **adaptor-related protein complex 5, beta 1 subunit** |
| **Milr1** | **1.944** | **mast cell immunoglobulin like receptor 1** |
| **Fmod** | **1.910** | **fibromodulin** |
| **Pald1** | **1.891** | **phosphatase domain containing, paladin 1** |
| **Fam178b** | **1.862** | **family with sequence similarity 178, member B** |
| **Fads2** | **1.862** | **fatty acid desaturase 2** |
| **Upb1** | **1.850** | **ureidopropionase, beta** |
| **Eme2** | **1.812** | **essential meiotic structure-specific endonuclease subunit 2** |
| **Naglu** | **1.790** | **alpha-N-acetylglucosaminidase (Sanfilippo disease IIIB)** |
| **Il34** | **1.784** | **interleukin 34** |
| **Socs4** | **1.767** | **suppressor of cytokine signaling 4** |
| **Cox4i2** | **1.761** | **cytochrome c oxidase subunit IV isoform 2** |
| **Fam69b** | **1.760** | **family with sequence similarity 69, member B** |
| **Nrg3** | **1.752** | **neuregulin 3** |
| **Bspry** | **1.743** | **B-box and SPRY domain containing** |
| **Rangrf** | **1.719** | **RAN guanine nucleotide release factor** |
| **Fam229b** | **1.709** | **family with sequence similarity 229, member B** |
| **Ttll1** | **1.701** | **tubulin tyrosine ligase-like 1** |
| **Adgre1** | **1.700** | **adhesion G protein-coupled receptor E1** |
| **Slc12a9** | **1.700** | **solute carrier family 12 (potassium /chloride transporters), member 9** |

**Table. 1. Up-regulated genes in embryonic salivary gland treated with NPY at E13.5**
