# Supplementary material for: Neuropeptides regulate embryonic salivary gland branching through the FGF/FGFR pathway in aging klotho‐deficient mice
Source: Aging Cell. 2024 Sep 6;23(12):e14329. doi: 10.1111/acel.14329 (PMC11634708; doi:10.1111/acel.14329)
Supplement: Supplementary file 2 — Table S2. [file ACEL-23-e14329-s005.docx]

| **Gene symbol** | **Fold change (FC)** | **Description** |
| --- | --- | --- |
| **Col25a1** | **0.257** | **collagen, type XXV, alpha 1** |
| **Gtse1** | **0.262** | **G two S phase expressed protein 1** |
| **4933400F21Rik** | **0.273** | **RIKEN cDNA 4933400F21 gene** |
| **Zdhhc8** | **0.298** | **zinc finger, DHHC domain containing 8** |
| **Magel2** | **0.317** | **melanoma antigen, family L, 2** |
| **Mettl10** | **0.317** | **methyltransferase like 10** |
| **Raf1** | **0.318** | **v-raf-leukemia viral oncogene 1** |
| **Bbs7** | **0.348** | **Bardet-Biedl syndrome 7 (human)** |
| **Plgrkt** | **0.358** | **plasminogen receptor, C-terminal lysine transmembrane protein** |
| **Snhg8** | **0.366** | **small nucleolar RNA host gene 8** |
| **Arhgap36** | **0.381** | **Rho GTPase activating protein 36** |
| **Bambi** | **0.386** | **BMP and activin membrane-bound inhibitor** |
| **Slc35e2** | **0.392** | **solute carrier family 35, member E2** |
| **Ptprm** | **0.401** | **protein tyrosine phosphatase, receptor type, M** |
| **Ldb1** | **0.403** | **LIM domain binding 1** |
| **Tsnax** | **0.408** | **translin-associated factor X** |
| **Trdmt1** | **0.410** | **tRNA aspartic acid methyltransferase 1** |
| **Itgb6** | **0.412** | **integrin beta 6** |
| **Snx3** | **0.416** | **sorting nexin 3** |
| **Rgmb** | **0.417** | **repulsive guidance molecule family member B** |
| **Zcchc14** | **0.422** | **zinc finger, CCHC domain containing 14** |
| **Lgi1** | **0.427** | **leucine-rich repeat LGI family, member 1** |
| **Hace1** | **0.429** | **HECT domain and ankyrin repeat containing, E3 ubiquitin protein ligase 1** |
| **Alk** | **0.435** | **anaplastic lymphoma kinase** |
| **Fat1** | **0.435** | **FAT atypical cadherin 1** |

**Table. 2. Down-regulated genes in embryonic salivary gland treated with NPY at E13.5**
