# Supplementary material for: Neuropeptides regulate embryonic salivary gland branching through the FGF/FGFR pathway in aging klotho‐deficient mice
Source: Aging Cell. 2024 Sep 6;23(12):e14329. doi: 10.1111/acel.14329 (PMC11634708; doi:10.1111/acel.14329)
Supplement: Supplementary file 3 — Table S3. [file ACEL-23-e14329-s006.docx]

| **Gene symbol** | **Fold change (FC)** | **Description** |
| --- | --- | --- |
| **Sez6** | **2.932** | **seizure related gene 6** |
| **Zfp273** | **2.384** | **zinc finger protein 273** |
| **Drd4** | **2.371** | **dopamine receptor D4** |
| **Ica1l** | **2.074** | **islet cell autoantigen 1-like** |
| **Fam178b** | **2.059** | **family with sequence similarity 178, member B** |
| **Ripk3** | **2.042** | **receptor-interacting serine-threonine kinase 3** |
| **Rab17** | **1.961** | **RAB17, member RAS oncogene family** |
| **Fmod** | **1.946** | **fibromodulin** |
| **Ston1** | **1.930** | **stonin 1** |
| **Rad52** | **1.925** | **RAD52 homolog, DNA repair protein** |
| **Gfra3** | **1.920** | **glial cell line derived neurotrophic factor family receptor alpha 3** |
| **1500015O10Rik** | **1.905** | **RIKEN cDNA 1500015O10 gene** |
| **Il34** | **1.867** | **interleukin 34** |
| **Hp** | **1.830** | **haptoglobin** |
| **Atp2c2** | **1.819** | **ATPase, Ca++ transporting, type 2C, member 2** |
| **9130011E15Rik** | **1.795** | **RIKEN cDNA 9130011E15 gene** |
| **Plekhf1** | **1.780** | **pleckstrin homology domain containing, family F (with FYVE domain) member 1** |
| **Tmem266** | **1.779** | **Transmembrane protein 266** |
| **Sept1** | **1.755** | **septin 1** |
| **Zbtb8b** | **1.744** | **zinc finger and BTB domain containing 8b** |
| **Foxo3** | **1.713** | **forkhead box O3** |
| **Fgf1** | **1.711** | **fibroblast growth factor 1** |
| **Tm9sf1** | **1.706** | **transmembrane 9 superfamily member 1** |
| **Upk2** | **1.685** | **uroplakin 2** |
| **Cdkl2** | **1.675** | **cyclin-dependent kinase-like 2 (CDC2-related kinase)** |

**Table. 3. Up-regulated genes in embryonic salivary gland treated with SP at E13.5**
