# Supplementary material for: Neuropeptides regulate embryonic salivary gland branching through the FGF/FGFR pathway in aging klotho‐deficient mice
Source: Aging Cell. 2024 Sep 6;23(12):e14329. doi: 10.1111/acel.14329 (PMC11634708; doi:10.1111/acel.14329)
Supplement: Supplementary file 4 — Table S4. [file ACEL-23-e14329-s004.docx]

| **Gene symbol** | **Fold change (FC)** | **Description** |
| --- | --- | --- |
| **Bdnf** | **0.418** | **brain derived neurotrophic factor** |
| **Angptl7** | **0.440** | **angiopoietin-like 7** |
| **Tmem179** | **0.441** | **transmembrane protein 179** |
| **Nrn1** | **0.446** | **neuritin 1** |
| **Kcnc1** | **0.448** | **potassium voltage gated channel, Shaw-related subfamily, member 1** |
| **Rab39b** | **0.448** | **RAB39B, member RAS oncogene family** |
| **1810062O18Rik** | **0.453** | **RIKEN cDNA 1810062O18 gene** |
| **C130046K22Rik** | **0.457** | **RIKEN cDNA C130046K22 gene** |
| **Fbxo41** | **0.458** | **F-box protein 41** |
| **Miat** | **0.471** | **myocardial infarction associated transcript (non-protein coding)** |
| **Itgb6** | **0.474** | **integrin beta 6** |
| **Zfp940** | **0.488** | **zinc finger protein 940** |
| **Kctd8** | **0.517** | **potassium channel tetramerisation domain containing 8** |
| **Dgke** | **0.518** | **diacylglycerol kinase, epsilon** |
| **Ppp4r1l-ps** | **0.519** | **protein phosphatase 4, regulatory subunit 1-like, pseudogene** |
| **Cdkl3** | **0.527** | **cyclin-dependent kinase-like 3** |
| **1700030J22Rik** | **0.528** | **RIKEN cDNA 1700030J22 gene** |
| **Tmem136** | **0.529** | **transmembrane protein 136** |
| **Gm20199** | **0.529** | **predicted gene, 20199** |
| **Zfp583** | **0.530** | **zinc finger protein 583** |
| **Il1rapl1** | **0.547** | **interleukin 1 receptor accessory protein-like 1** |
| **Bhlhe41** | **0.558** | **basic helix-loop-helix family, member e41** |
| **Acan** | **0.558** | **aggrecan** |
| **Trank1** | **0.566** | **tetratricopeptide repeat and ankyrin repeat containing 1** |
| **Chrd** | **0.568** | **chordin** |

**Table. 4. Down-regulated genes in embryonic salivary gland treated with of SP at E13.5**
