# Supplementary figures and images for: Neuropeptides regulate embryonic salivary gland branching through the FGF/FGFR pathway in aging klotho‐deficient mice
Source: Aging Cell. 2024 Sep 6;23(12):e14329. doi: 10.1111/acel.14329 (PMC11634708; doi:10.1111/acel.14329)

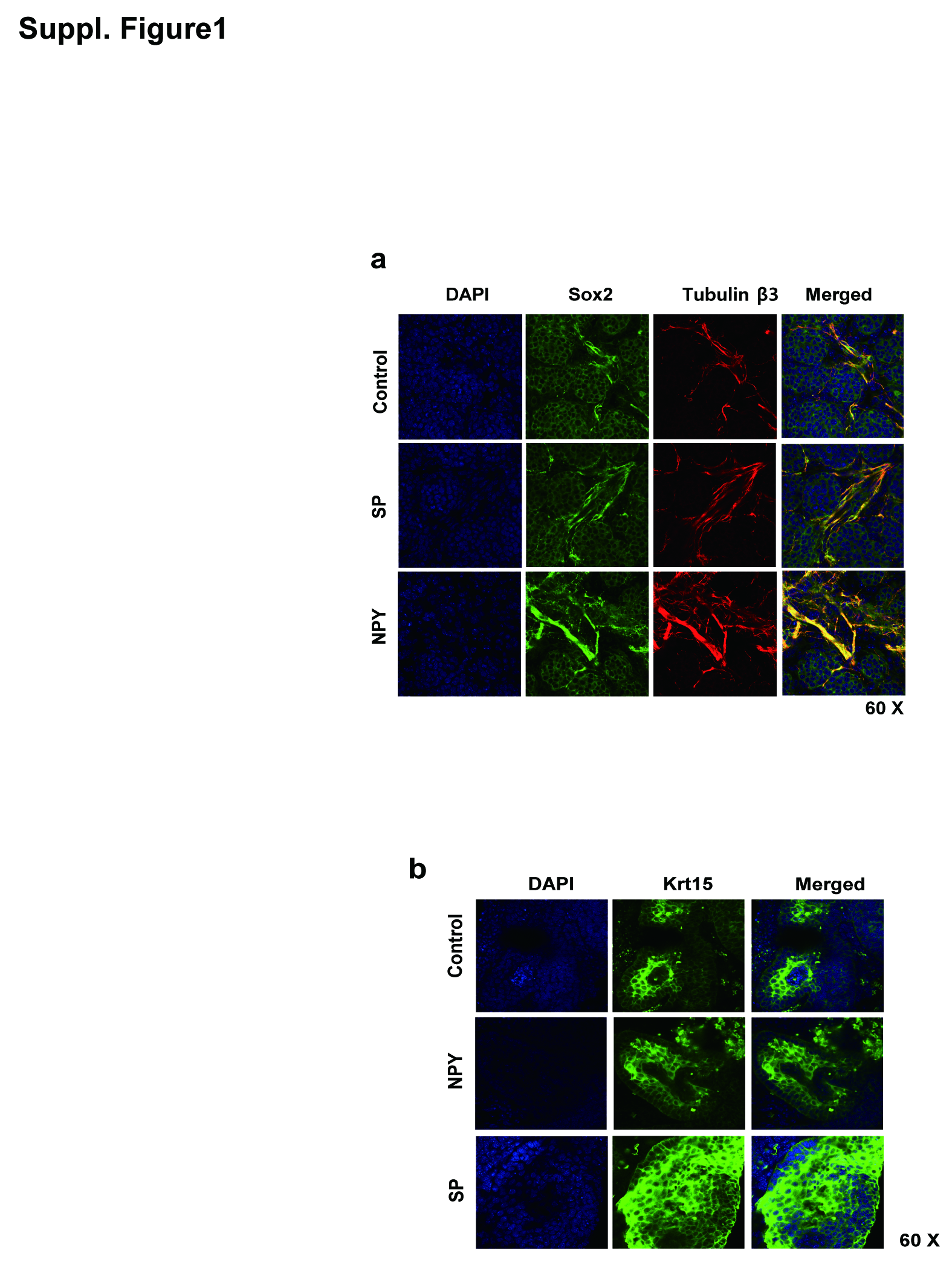

Supplement: Supplementary file 5 — Figure S1. Immunostaining for Sox2/Tubb3 and Krt15. (a) Immunostaining of the parasympathetic nervous marker tubulin β3 and stem cell markers Sox2, using confocal microscopy. 60× magnification of Figure 2d. (b) Immunostaining with Krt15 after neuropeptide treatment. [file ACEL-23-e14329-s001.tif]

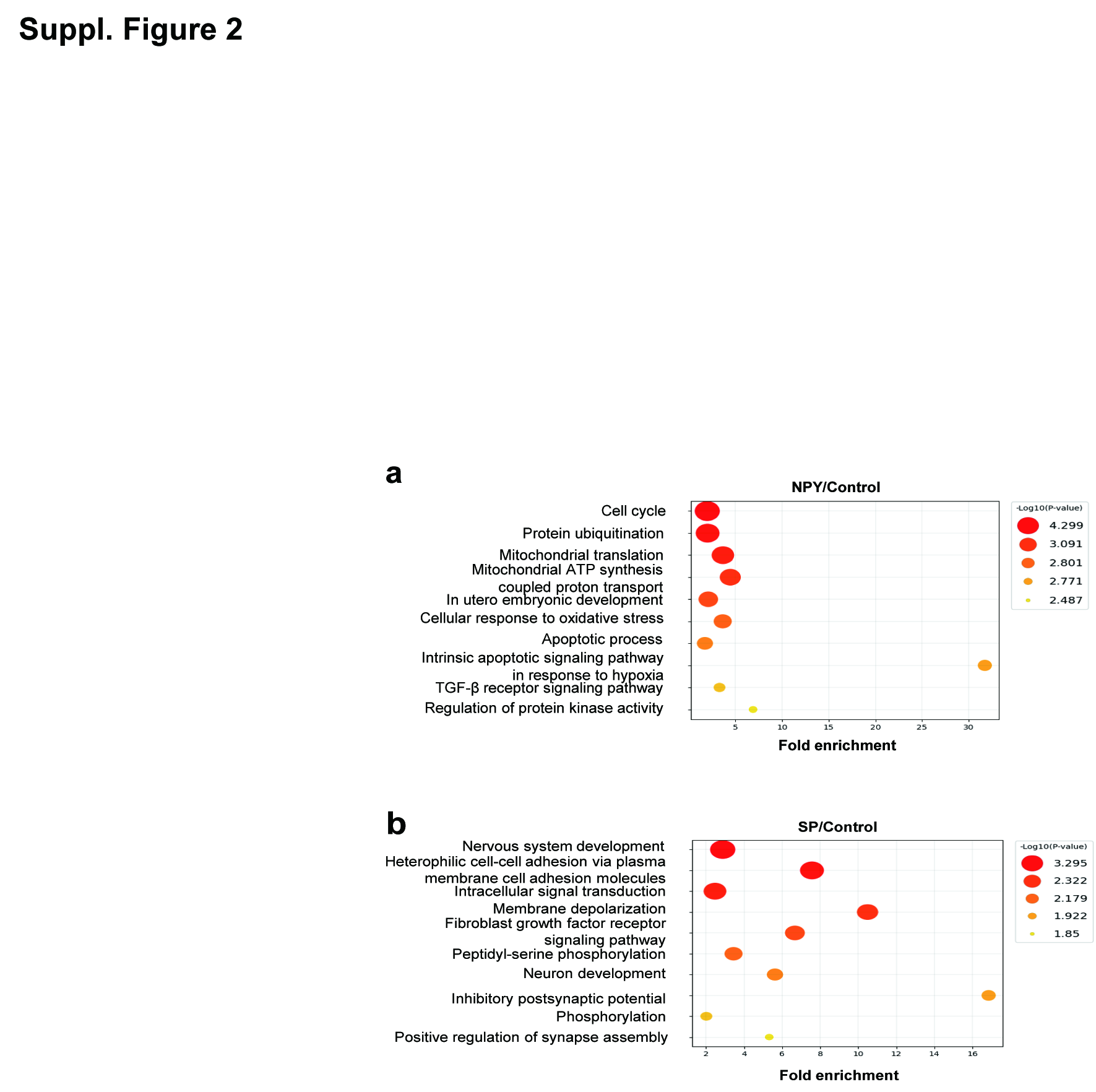

Supplement: Supplementary file 6 — Figure S2. RNA‐seq profiling. (a,b) Gene Ontology enrichment analysis of biological process terms between the NPY‐ and SP‐treated groups versus control samples generated from the DAVID database. All the figures were generated using ExDEGA software from Ebiogen. [file ACEL-23-e14329-s002.tif]

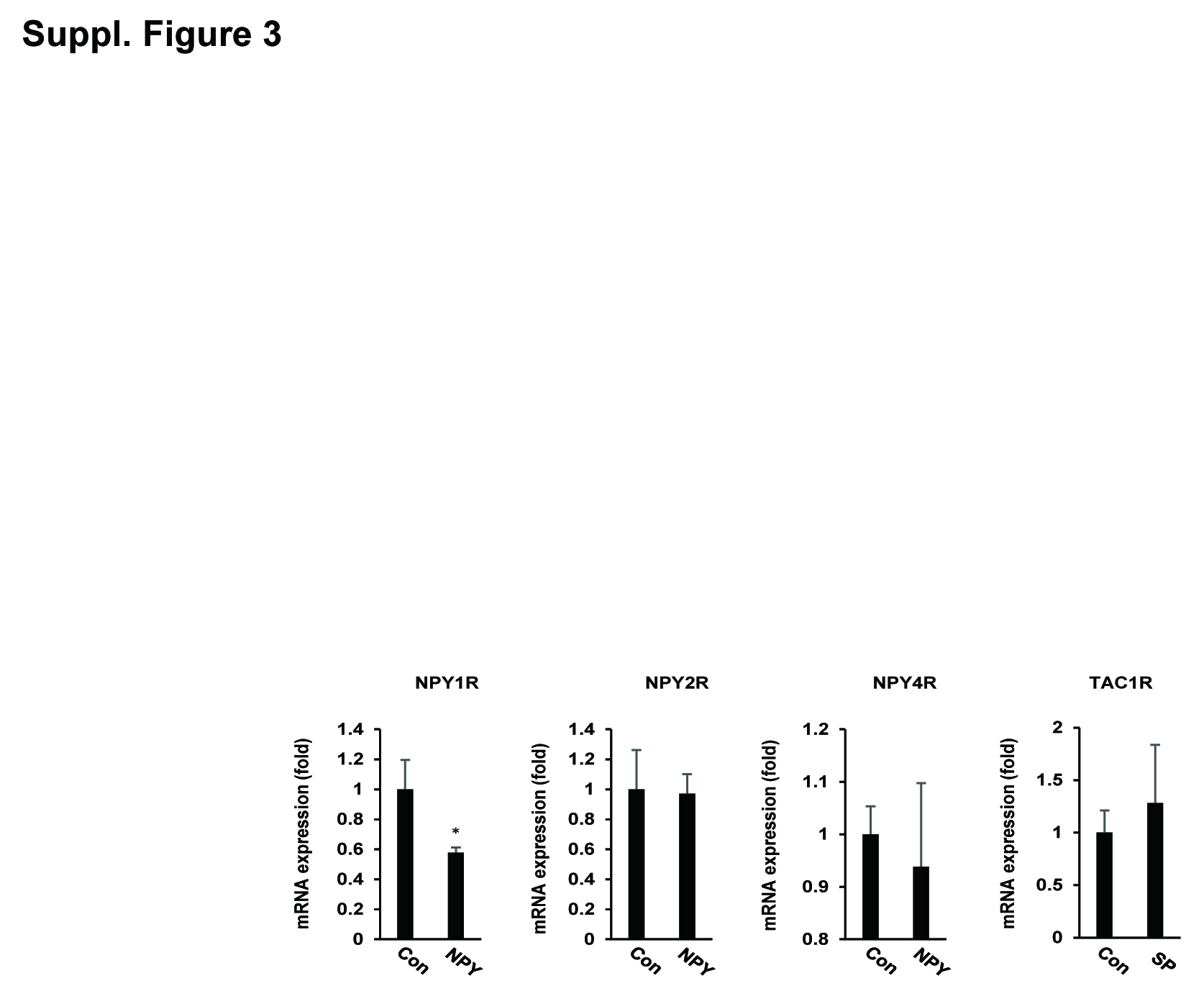

Supplement: Supplementary file 7 — Figure S3. qRT‐PCR analysis of the specific receptors against NPY and SP. NPY, neuropeptide Y; PCR, polymerase chain reaction; qRT‐PCR, quantitative real‐time PCR; SP, substance P. [file ACEL-23-e14329-s003.tif]
